# Supplementary material for: Genetic and dietary determinants of gut microbiome-bile acid interactions in the BXD genetic reference population
Source: Nat Commun. 2025 Dec 18;17:956. doi: 10.1038/s41467-025-67680-x (PMC12848039; doi:10.1038/s41467-025-67680-x)
Supplement: Supplementary file 1 — Supplementary Information [file 41467_2025_67680_MOESM1_ESM.pdf]

Genetic and dietary determinants of gut microbiome-bile acid interactions in the BXD genetic reference population

Xiaoxu Li[1], Alessia Perino[2], Jonathan Sulc[1], Antoine Jalil[2], Giacomo V.G. von Alvensleben[1], Jean-David Morel[1], Qi Wang[1], Alexis Rapin[1], Hao Li[1,3], Kristina Schoonjans[2,*], Johan Auwerx[1,*]

[1]Laboratory of Integrative Systems Physiology, Institute of Bioengineering, École Polytechnique Fédérale de Lausanne, 1015 Lausanne, Switzerland.

[2]Laboratory of Metabolic Signaling, Institute of Bioengineering, École Polytechnique Fédérale de Lausanne, 1015 Lausanne, Switzerland.

[3]Center for Mitochondrial Biology and Medicine, The Key Laboratory of Biomedical Information Engineering of Ministry of Education, School of Life Science and Technology, Xi'an Jiaotong University, Xi'an, China

*e-mail for correspondence: kristina.schoonjans@epfl.ch (KS) or admin.auwerx@epfl.ch (JA)

Supplementary Figures:

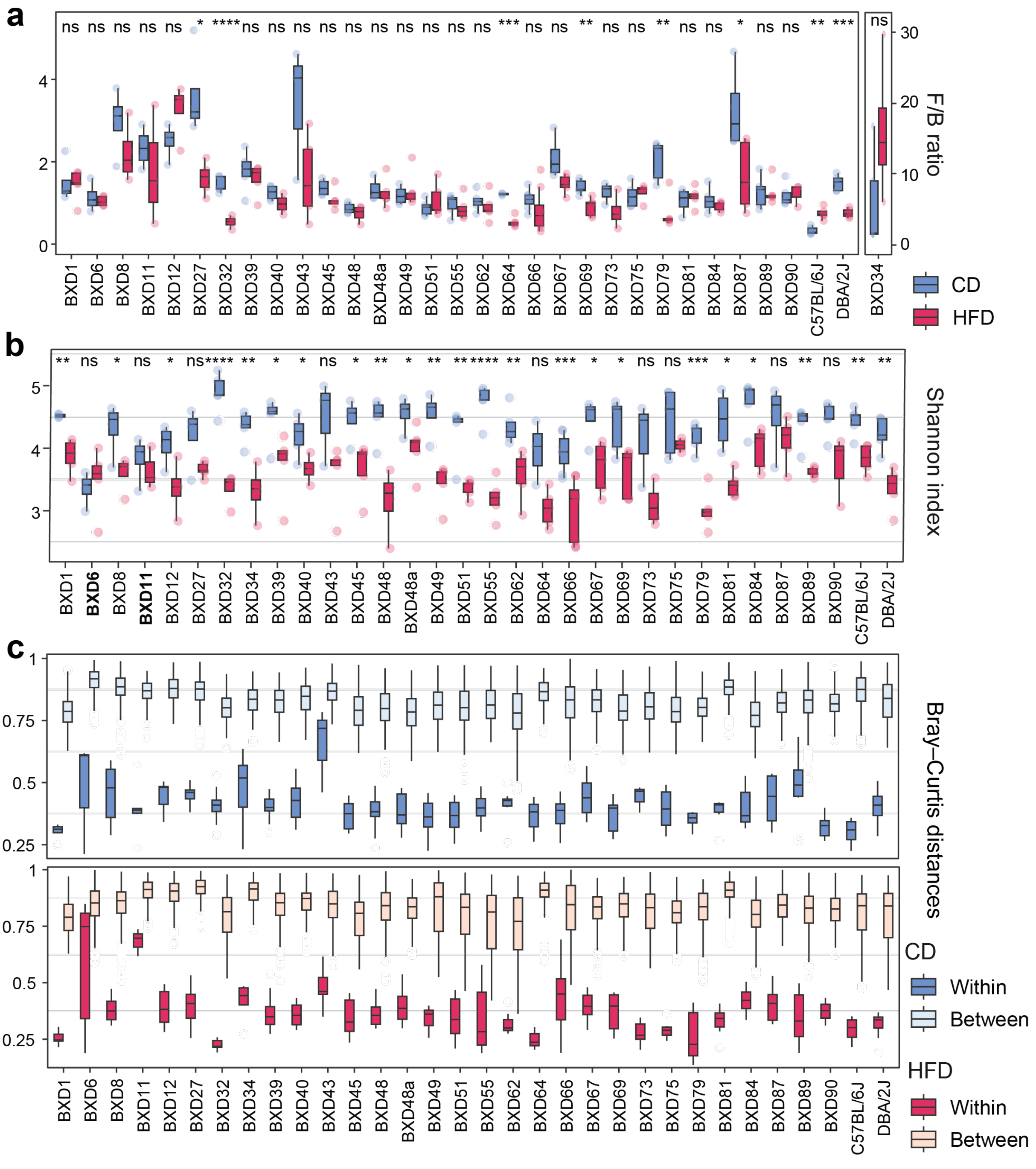

Supplementary Figure 1: Alpha and beta diversity analyses of the BXD gut microbiome. (**a**) The ratio of Firmicutes to Bacteroidetes (F/B ratio) in the cecum of each BXD strain fed with CD or HFD. (**b**) Cecal bacterial community diversity in terms of Shannon index in the cecum of each BXD strain fed CD and HFD. (**c**) Box plots showing Bray–Curtis distances of cecal microbial communities within each BXD strain (individual differences) and between strains (genetic differences) under CD and HFD. P values were calculated by two-tailed Student's t-test and indicated as follows: *P value <0.05; **P value < 0.01; ***P value < 0.001;

****P value < 0.0001. ns: not significant. Box plots characterize samples using the lower quartile, median, and the upper quartile. Source data are provided as a Source Data file.

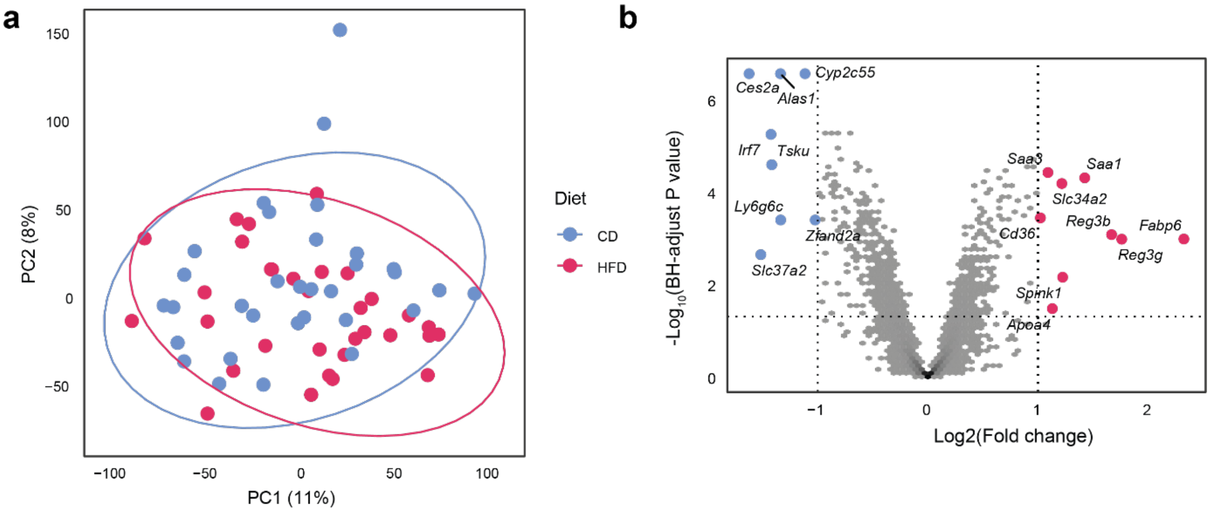

Supplementary Figure 2: HFD effect on the host gene expression in BXD colons. (**a**) Principal component analysis (PCA) of the microarray profiles of BXD colons under CD and HFD. (**b**) Volcano plot showing the HFD effect on BXD colon transcriptome and the up- and down-regulated differentially expressed genes (DEGs, absolute Log2(Fold change) > 1 and BH-adjusted P value < 0.05). Source data are provided as a Source Data file.

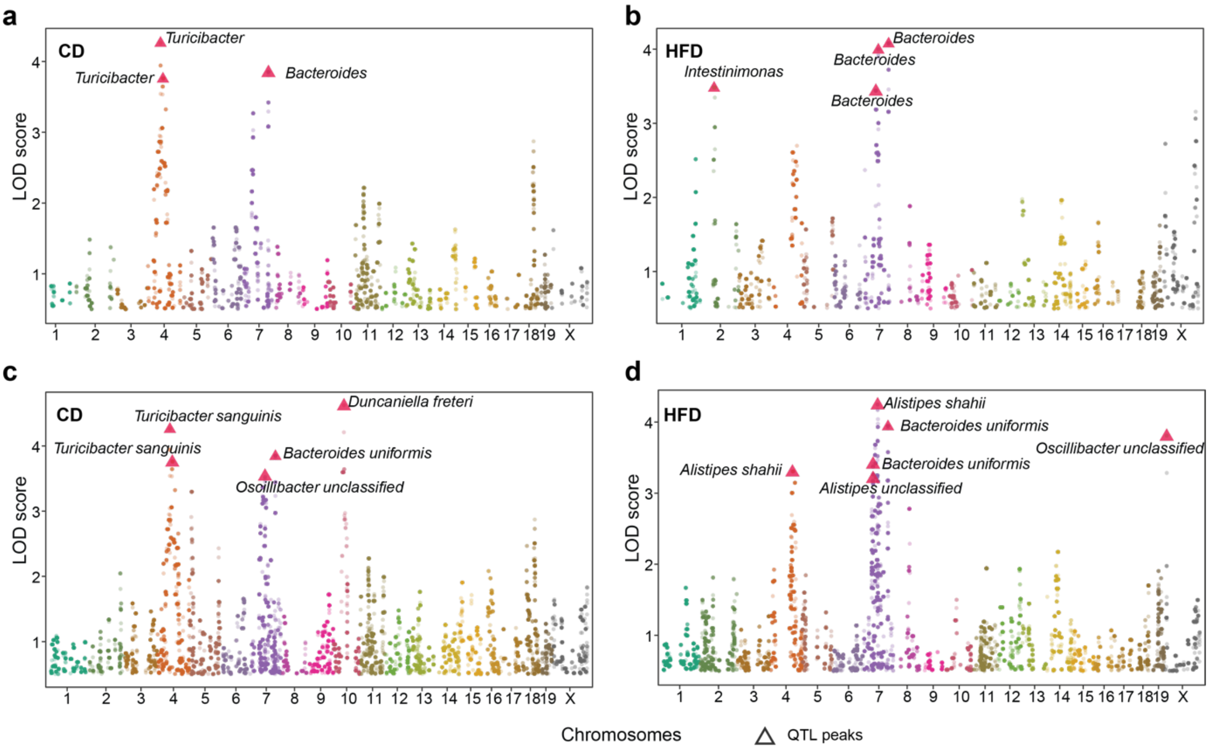

Supplementary Figure 3: QTL mapping analyses of gut bacterial genera and species in the BXDs. (**a**, **b**) Manhattan plots showing the associations between genetic loci and bacterial genera under CD (**a**) and HFD (**b**). (**c**, **d**) Manhattan plots showing the associations between genetic loci and bacterial species under CD (**c**) and HFD (**d**). The genetic locus-bacterium associations with LOD > 0.5 are shown. Each chromosome is indicated by color and the significant genetic locus-bacterium associations are represented by triangles. Source data are provided as a Source Data file.

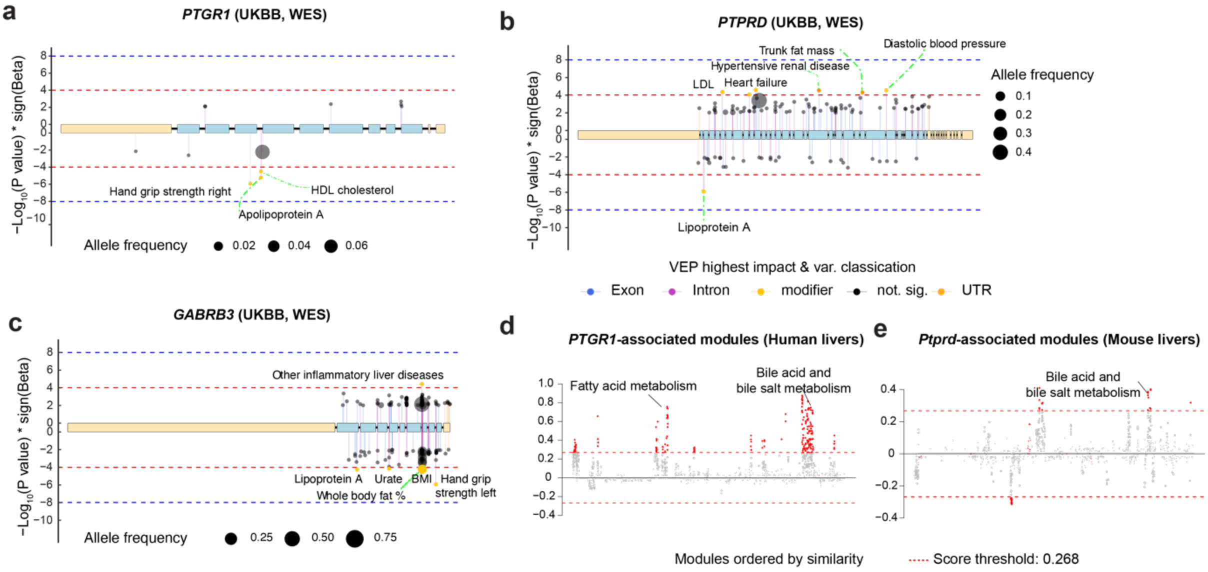

Supplementary Figure 4: Candidate gene-BA metabolism module associations. (**a-c**) Lollipop plots showing the associations between metabolic traits and genetic variants in *PTGR1* (**a**), *PTPRD* (**b**), and *GABRB3* (**c**) obtained from GWAS results in the European population of the UKBB based on the whole exome sequence (WES) data[55]. Only suggestive ($-Log_{10}$(P value) > 4, red dashed line) or significant ($-Log_{10}$(P value) > 8, blue dashed line) genetic variant-phenotype associations are indicated. Variant effect was predicted, and their classifications are represented by colors. Allele frequency of genetic variants is indicated by dot size. VEP: Variant Effect Prediction. (**d**, **e**) Manhattan plots showing the associated gene expression modules of Prostaglandin Reductase 1 (*PTGR1)* in human livers (**d**) and that of Protein Tyrosine Phosphatase Receptor Type D (*Ptprd)* in mouse livers (**e**). Data were retrieved from https://systems-genetics.org/gmad. The threshold is represented by the red dashed line (absolute gene-module association score ≥ 0.268). Terms above the threshold are identified as the significant associated terms. GO terms or gene modules are ranked by similarity. Source data are provided as a Source Data file.

Supplementary Table 1. List of the BAs measured, their abbreviations, accurate m/z ratio, corresponding internal standards (used for quantification) and retention times (RT). Chromatographic resolution was essential for the separation of isobaric species.

Bile Acid	Abbreviation	H^- (m/z)	Internal Standard	RT min
Lithocholic acid	LCA	375.29047	d_4-LCA	20.6
7-Ketolithocholic acid	7Keto-LCA	389.26973	d_4-GDCA	15.5
Murocholic acid	MDCA	391.28538	d_4-CA	12.4
Ursodeoxycholic acid	UDCA	391.28538	d_4-GDCA	13.3
Chenodeoxycholic acid	CDCA	391.28538	d_4-CDCA	18.3
Deoxycholic acid	DCA	391.28538	d_4-DCA	18.5
Isodeoxycholic acid	isoDCA	391.28538	d_4-GDCA	19.7
3-Oxocholic acid	3Oxo-CA	405.26465	d_4-TCDCA	12.6
7-Ketodeoxycholic acid	7Keto-DCA	405.26465	d_4-GCA	11.0
α-Muricholic acid	α-MCA	407.28030	d_4-GCA	10.8
β-Muricholic acid	β-MCA	407.28030	d_4-GCA	11.2
ω-Muricholic acid	ω-MCA	407.28030	d_4-GCA	10.4
γ-Muricholic acid	MCA	407.28030	d_4-GCA	11.8
Cholic acid	CA	407.28030	d_4-CA	12.6
Glycolithocholic acid	GLCA	432.31193	d_4-GDCA	13.5
Glycoursodeoxycholic acid	GUDCA	448.30685	d_4-GCA	10.2
Glycohyodeoxycholic acid	GHDCA	448.30685	d_4-CA	10.6
Glycochenodeoxycholic acid	GCDCA	448.30685	d_4-GCDCA	12.9
Glycocholic acid	GCA	464.30176	d_4-GCA	10.3
Taurolithocholic acid	TLCA	482.29457	d_5-TLCA	13.0
Tauroursodeoxycholic acid	TUDCA	498.28948	d_4-TCA	5.1
Taurohyodeoxycholic acid	THDCA	498.28948	d_4-TCDCA	5.5
Taurochenodeoxycholic acid	TCDCA	498.28948	d_4-TCDCA	10.6
Taurodeoxycholic acid	TDCA	498.28948	d_5-TDCA	10.9
Tauro-α-muricholic acid	TαMCA	514.28440	d_4-TαMCA	2.1
Tauro-β-muricholic acid	TβMCA	514.28440	d_4-TβMCA	2.3
Taurocholic acid	TCA	514.28440	d_4-TCA	5.8

Supplemental table 2. QTL peaks of both bacterial abundance and BA profiles within identified gMxBs under CD and HFD, related to Fig.5a-d and Table 1.

gMxBs	Phenotype	Chr	LOD	Diet	Type	QTL peak (Start position)	QTL peak (End position)
gMxB1	*Turicibacter*	4	4.27	CD	Microbiome	46.346673	68.274947
	Turicibacter sanguinis	4	4.27	CD	Microbiome	46.346673	68.274947
	CA/All (Liver)	4	3.8	CD	BA	62.608978	96.668598
	CA (Plasma T0)	4	3.78	CD	BA	40.278465	65.944235
	CDCA (Plasma T0)	4	3.61	CD	BA	31.419721	65.63108202
	CA/All (Plasma T0)	4	3.56	CD	BA	32.081863	65.944235
	TCA/All (Plasma T0)	4	3.99	CD	BA	27.800645	53.142706
	TCA/All (Plasma T30)	4	3.69	CD	BA	19.574375	54.898634
gMxB2	*Turicibacter*	4	3.76	CD	Microbiome	68.274947	88.039053
	Turicibacter sanguinis	4	3.76	CD	Microbiome	68.274947	88.039053
	CA/All (Liver)	4	3.8	CD	BA	62.608978	96.668598
gMxB3	*Bacteroides uniformis*	7	3.42	HFD	Microbiome	40.918795	46.449614
	α-MCA (Feces)	7	3.78	HFD	BA	41.954198	55.607957
	TLCA (Feces)	7	3.63	HFD	BA	40.386476	46.449614
	UDCA (Feces)	7	3.62	HFD	BA	42.214611	55.591032
gMxB4	*Bacteroides*	7	3.44	HFD	Microbiome	55.653463	66.326665
	7-keto-LCA (Feces)	7	3.19	HFD	BA	46.855159	62.20968
	7-keto-DCA/All (Feces)	7	3.40	HFD	BA	46.855159	62.209688
