# Supplementary material for: Genetic and dietary determinants of gut microbiome-bile acid interactions in the BXD genetic reference population
Source: Nat Commun. 2025 Dec 18;17:956. doi: 10.1038/s41467-025-67680-x (PMC12848039; doi:10.1038/s41467-025-67680-x)
Supplement: Supplementary file 2 — Description Of Additional Supplementary File [file 41467_2025_67680_MOESM2_ESM.pdf]

Description of Additional supplementary files

Supplementary Data1. The list of instrumental variables used in the Mendelian randomization analysis to evaluate the causal effect of PTGR1 gene expression in the human colon on the abundance of Turicibacter, related to Fig.6b
