# Supplementary material for: Genetic and dietary determinants of gut microbiome-bile acid interactions in the BXD genetic reference population
Source: Nat Commun. 2025 Dec 18;17:956. doi: 10.1038/s41467-025-67680-x (PMC12848039; doi:10.1038/s41467-025-67680-x)
Supplement: Supplementary file 4 — Reporting Summary [file 41467_2025_67680_MOESM4_ESM.pdf]

nature portfolio

Corresponding author(s): Kristina Schoonjans & Johan Auwerx

Last updated by author(s): Nov 24, 2025

Reporting Summary

Nature Portfolio wishes to improve the reproducibility of the work that we publish. This form provides structure for consistency and transparency in reporting. For further information on Nature Portfolio policies, see our Editorial Policies and the Editorial Policy Checklist.

Statistics

For all statistical analyses, confirm that the following items are present in the figure legend, table legend, main text, or Methods section.

n/a	Confirmed	
☐	☒	The exact sample size (*n*) for each experimental group/condition, given as a discrete number and unit of measurement
☐	☒	A statement on whether measurements were taken from distinct samples or whether the same sample was measured repeatedly
☐	☒	The statistical test(s) used AND whether they are one- or two-sided<br>*Only common tests should be described solely by name; describe more complex techniques in the Methods section.*
☐	☒	A description of all covariates tested
☐	☒	A description of any assumptions or corrections, such as tests of normality and adjustment for multiple comparisons
☐	☒	A full description of the statistical parameters including central tendency (e.g. means) or other basic estimates (e.g. regression coefficient) AND variation (e.g. standard deviation) or associated estimates of uncertainty (e.g. confidence intervals)
☐	☒	For null hypothesis testing, the test statistic (e.g. *F*, *t*, *r*) with confidence intervals, effect sizes, degrees of freedom and *P* value noted<br>*Give P values as exact values whenever suitable.*
☒	☐	For Bayesian analysis, information on the choice of priors and Markov chain Monte Carlo settings
☒	☐	For hierarchical and complex designs, identification of the appropriate level for tests and full reporting of outcomes
☐	☒	Estimates of effect sizes (e.g. Cohen's *d*, Pearson's *r*), indicating how they were calculated

Our web collection on statistics for biologists contains articles on many of the points above.

Software and code

Policy information about availability of computer code

Data collection	The following software were used for data collection:<br>Illumina MiSeq platform for gut microbiome;<br>Direct-zol (Zymo Research), Ambion® WT Expression Kit, Affymetrix WT terminal labeling kit, and Affymetrix mouse Clariom S Assay for microarray data.
Data analysis	The following software were used for data analysis:<br>r studio (v.4.4.2);<br>Illumina-utils python package (version 2.7);<br>dada2 R package (version 1.18);<br>vegan R package (version 2.6-4);<br>Phyloseq R package (version 1.38.0);<br>ANCOMBC R package (version 2.4.0);<br>Array Power Tool (APT) suite (v2.11.3);<br>FactoMineR package (version 2.9);<br>limma R package (version 3.50.3);<br>PMA R package (version 1.2-2);<br>clusterProfiler R package (version 4.2.2);<br>msigdbr R package (version 7.5.1);<br>glmnet R package (version 4.1-8);<br>qtl2 R package (version 0.36);

REGENIE;
TwoSampleMR R package (version 0.6.9)

For manuscripts utilizing custom algorithms or software that are central to the research but not yet described in published literature, software must be made available to editors and reviewers. We strongly encourage code deposition in a community repository (e.g. GitHub). See the Nature Portfolio guidelines for submitting code & software for further information.

Data

Policy information about availability of data

All manuscripts must include a data availability statement. This statement should provide the following information, where applicable:

- Accession codes, unique identifiers, or web links for publicly available datasets
- A description of any restrictions on data availability
- For clinical datasets or third party data, please ensure that the statement adheres to our policy

The colon transcriptome data generated in this study have been deposited in the GEO database under accession code: GSE272489 (https://www.ncbi.nlm.nih.gov/geo/query/acc.cgi?acc=GSE272489). The cecal 16S ribosomal RNA sequencing generated in this study have been deposited in the NCBI Sequence Read Archive (SRA) under accession code PRJNA1137099 (https://www.ncbi.nlm.nih.gov/sra?linkname=bioproject_sra_all&from_uid=1137099). The phenotype and bile acid data used in this study are available in the Mouse Phenome Database: https://phenome.jax.org/projects/Schoonjans1. Source Data are provided with this paper.

Research involving human participants, their data, or biological material

Policy information about studies with human participants or human data. See also policy information about sex, gender (identity/presentation), and sexual orientation and race, ethnicity and racism.

Reporting on sex and gender	This study only considered sex in the data analyses and this information was provided by the UK Biobank.
Reporting on race, ethnicity, or other socially relevant groupings	The population of European descent was used in this study and identified by the UKBB return dataset 2442
Population characteristics	The population characteristics were provided by the UK Biobank. Age and sex were used as covariants in the GWAS analysis
Recruitment	Individuals were recruited by the UK Biobank.
Ethics oversight	We have been allowed to use the UK Biobank Resource under Application Number 48020

Note that full information on the approval of the study protocol must also be provided in the manuscript.

Field-specific reporting

Please select the one below that is the best fit for your research. If you are not sure, read the appropriate sections before making your selection.

☒ Life sciences ☐ Behavioural & social sciences ☐ Ecological, evolutionary & environmental sciences

For a reference copy of the document with all sections, see nature.com/documents/nr-reporting-summary-flat.pdf

Life sciences study design

All studies must disclose on these points even when the disclosure is negative.

Sample size	No statistical method was used to determine sample size. Sample size was based on our previous published measurements of bile acids. (Cell metabolism, 34(10), 1594–1610.e4.)
Data exclusions	To confirm the quality of 16S data, samples with less than 6,000 sequencing reads (2 samples) were discarded for the microbiome analysis
Replication	The in vivo study was performed once, but included biological replicates. The mice used for experiments were from multiple different litters.
Randomization	Mice were randomly assigned into chow or high-fat diet-feeding condition, controlling for initial body weight.
Blinding	The investigators were not blinded during data collection. Computational analysis was not performed blinded.

Reporting for specific materials, systems and methods

We require information from authors about some types of materials, experimental systems and methods used in many studies. Here, indicate whether each material, system or method listed is relevant to your study. If you are not sure if a list item applies to your research, read the appropriate section before selecting a response.

Materials & experimental systems

n/a	Involved in the study
☒	☐ Antibodies
☒	☐ Eukaryotic cell lines
☒	☐ Palaeontology and archaeology
☐	☒ Animals and other organisms
☒	☐ Clinical data
☒	☐ Dual use research of concern
☒	☐ Plants

Methods

n/a	Involved in the study
☒	☐ ChIP-seq
☒	☐ Flow cytometry
☒	☐ MRI-based neuroimaging

Animals and other research organisms

Policy information about studies involving animals; ARRIVE guidelines recommended for reporting animal research, and Sex and Gender in Research

Laboratory animals	30 BXD strains (BXD1, BXD11, BXD12, BXD27, BXD32, BXD34, BXD39, BXD40, BXD43, BXD45, BXD48, BXD48a, BXD49, BXD51, BXD55, BXD6, BXD62, BXD64, BXD66, BXD67, BXD69, BXD73, BXD75, BXD79, BXD8, BXD81, BXD84, BXD87, BXD89, BXD90, C57BL/6J, DBA/2J) and 2 parental strains (C57BL/6J and DBA/2J) were included
Wild animals	Study did not involve wild animals
Reporting on sex	Male mice were used in this study to limit the number of animals.
Field-collected samples	Study did not involve field-collected samples, All mice were housed under 12 h light/dark cycle (lights on at 7am), with a temperature of 22°C ± 1°C with 30–50% humidity.
Ethics oversight	All animal experiments comply with all relevant ethical regulations and they were approved by the Swiss cantonal veterinary authorities of Vaud under the license 2257.2.

Note that full information on the approval of the study protocol must also be provided in the manuscript.

Plants

Seed stocks	*Report on the source of all seed stocks or other plant material used. If applicable, state the seed stock centre and catalogue number. If plant specimens were collected from the field, describe the collection location, date and sampling procedures.*
Novel plant genotypes	*Describe the methods by which all novel plant genotypes were produced. This includes those generated by transgenic approaches, gene editing, chemical/radiation-based mutagenesis and hybridization. For transgenic lines, describe the transformation method, the number of independent lines analyzed and the generation upon which experiments were performed. For gene-edited lines, describe the editor used, the endogenous sequence targeted for editing, the targeting guide RNA sequence (if applicable) and how the editor was applied.*
Authentication	*Describe any authentication procedures for each seed stock used or novel genotype generated. Describe any experiments used to assess the effect of a mutation and, where applicable, how potential secondary effects (e.g. second site T-DNA insertions, mosiacism, off-target gene editing) were examined.*
